# Survey data of determinants related to Covid-19 preventive behaviors during the second waves in Indonesia using the reasoned action approach

**DOI:** 10.1016/j.dib.2022.108147

**Published:** 2022-04-09

**Authors:** Triana Kesuma Dewi, Dyah Ayu Savitri, Muchlisah Audina Sudirman, Ratri Aisyah Rohmatullaili, Shafira Rahmadianti, Rahkman Ardi

**Affiliations:** Faculty of Psychology, Universitas Airlangga, Jl. Airlangga 4-6, Surabaya, East Java 60286, Indonesia

**Keywords:** Covid-19 pandemic, Distancing, Good health and well-being, Hand washing, Social determinants of health, Mask use

## Abstract

The dataset presented in this article examines preventive behaviors related to Covid-19 among Indonesians, i.e., wearing a mask, hand washing, and distancing, and its related determinants. Data collection was carried out when Indonesia was facing the second waves due to the rapid transmission of the Delta variant virus. The data assessing socio-demographic information, beliefs related to Covid-19 preventive behaviors based on the reasoned action approach, general beliefs about Covid-19, and Covid-19 preventive behaviors, from 18th June to 18th August 2021, gathering a total of 863 completed responses. The sampling technique in this online survey used a combination of purposive and snowball sampling methods to gather the participants via social media such as Instagram, Twitter, Facebook, and WhatsApp throughout Indonesia. The survey data were analyzed using frequency distributions and bivariate correlation analysis. The data will help to understand the psychosocial determinants of Covid-19 preventive behaviors and provide insight for the development of behavior change intervention to limit the spread of Covid-19 among Indonesians.

## Specifications Table

**Table:** 

Subject	Public Health
Specific subject area	Health Psychology, Social Psychology
Type of data	Primary data, Tables
How the data were acquired	The data were collected using an online survey platform (google form). The questionnaires are included in this article and can be accessed via Open Science Framework (OSF)
Data format	Raw, Analyzed, Filtered (descriptive and inferential statistics)
Description of data collection	Survey data were obtained from 863 Indonesian participants aged 18 years old and older with internet access throughout Indonesia.
Data source location	Region: Asia Country: Indonesia
Data accessibility	Repository name: Open Science Framework (OSF) Data identification number (permanent identifier, i.e., DOI number): 10.17605/OSF.IO/89FG3 Direct link to the dataset: https://osf.io/89fg3/

## Value of the Data

• The data represent the first exploration of psychosocial determinants of Covid-19 preventive behaviors during the second waves from all 34 provinces in Indonesia.
• The data provide information about the development of determinants related to Covid-19 preventive behaviors (i.e., wearing a mask, hand washing, and distancing) scales based on the reasoned action approach.
• The data will be useful for researchers who want to compare with similar studies on determinants of Covid-19 preventive behaviors from other countries around the world and may serve as a heuristic basis for further insight to understand Covid-19 preventive behaviors.
• The data can be statistically analyzed to examine the relationship between socio-demographic information, reasoned action approach sub-constructs, and Covid-19 preventive behaviors.
• The data will be useful for researchers, practitioners, or policy-makers who want to develop an experiment or design an intervention plan to enhance Covid-19 preventive behaviors among Indonesians, e.g., design an experiment using question behavior effect [1] to promote Covid-19 preventive behaviors.

## 1 Data Description

The data set provides information on survey data of Covid-19 preventive behaviors using the reasoned action approach (RAA) [2] among Indonesians. We used three sets of online questionnaires related to Covid-19 preventive behaviors, including wearing a mask (*n* = 347), hand washing (*n* = 299), and distancing (*n* = 217). The obtained raw data used for each table is stored in a Microsoft Excel Worksheet (xls). Socio-demographic information includes age, education, employment, monthly family income, and history of Covid-19. Covid-19 preventive behaviors were measured with a single measure (i.e., “Over the past week, I … worn a mask when leaving home”), to elicit the response: almost never (less than 20%), rarely (21–40%), sometimes (41–60%), often (61–80%), or almost always (81–100%), which is further, for descriptive purpose, grouped into less frequent (up to 40%) and more frequent (more than 40%). We developed the measurement of determinants related to Covid-19 preventive behaviors based on the reasoned action approach (RAA) framework. Each item was rated using a 7-point Likert scale ranging from 1 = strongly disagree to 7 = strongly agree; and 1 = very unlikely can control to 7 = very likely can control, for perceived autonomy scales. Additionally, general beliefs about Covid-19 were also assessed with five items of 7-point Likert scale (e.g., “Corona virus and Covid-19 is …”, to elicit the response 1 = hoax to 7 = real). A detailed questionnaire will be provided as supplementary materials.

A. Wearing a mask

The measurement of determinants related to wearing a mask were grouped into experiential attitude (13 items, α = 0.90, e.g., “Wearing a mask when leaving the house during the pandemic makes me feel safer”), instrumental attitude (9 items, α = 0.98, e.g., “Wearing a mask when leaving the house during the pandemic prevents me from getting infected by Covid-19 Virus”), descriptive norms (14 items, α = 0.95, e.g., “My family members wear a mask when leaving the house during the pandemic”), injunctive norms (15 items, α = 0.94, e.g., “My partner expects me to always wear a mask when leaving the house during the pandemic”), perceived capacity (12 items, α = 0.96, e.g., “I believe I can wear a mask when shopping at the store/supermarket/market during the pandemic”), perceived autonomy (13 items, α = 0.96, e.g., “How far you can control yourself to keep wearing a mask when shopping at the store/supermarket/market during the pandemic”), and intentions (4 items, α = 0.88, e.g., “I intend to wear a mask when leaving the house during the pandemic”). Most of the participants (93.6%) reported wearing a mask more frequently during the past week. Detailed information about the socio-demographic difference among participants who less vs. more frequently wearing a mask and psychosocial determinants’ mean difference are shown in Tables 1 and 2, respectively. The correlations among study variables related to wearing a mask were reported in Table 3.

**Table 1:** Sociodemographic characteristics of the participants for wearing a mask.

	Less frequent		More frequent		
	N	%	N	%	Statistics
**Age**					*χ^2^*(1) = .39, *p* = .53, *V* = .03
18–30	16	4.6	255	73.5	
31–65	6	1.7	70	20.2	
**Education**					*χ^2^*(1) = 1.16, *p* = .28, *V* = .06
Lower education (up to senior high school)	12	3.5	139	40.1	
Higher Education (university)	10	2.9	186	53.6	
**Employment**					*χ^2^*(2) = 1.17, *p* = .56, *V* = .06
Unemployment, student, WFH	15	4.3	218	62.8	
WFO	4	1.2	81	23.3	
Health professional	3	.9	26	7.5	
**Family monthly income**					*χ^2^*(1) = .01, *p* = .98, *V* = .01
Below national standard	12	3.5	178	51.3	
National standard and above	10	2.9	147	42.4	
**History of Covid-19**					*χ^2^*(1) = 2.96, *p* = .08, *V* = .09
Yes	6	1.7	45	13	
No	16	4.6	280	80.7

**Table 2:** Comparison of RAA determinants and general beliefs for wearing a mask.

	Less frequent	More frequent	Statistics
	Mean (SD)	Mean (SD)	
Intention	6.56 (1.31)	6.79 (.51)	*t*(345) = −1.77, *p* = .08, *d* = .23
Instrumental attitude	6.08 (1.50)	6.24 (1.17)	*t*(345) = −.62, *p* = .53, *d* = .09
Experiential attitude	5.9 (1.33)	6.05 (.91)	*t*(345) = −.73, *p* = .47, *d* = .12
Injunctive Norms	6.10 (1.24)	6.30 (.76)	*t*(345) = −1.14, *p* = .25, *d* = .19
Descriptive Norms	5.84 (1.46)	6.15 (.79)	*t*(345) = −1.65, *p* = .10, *d* = .22
PBC autonomy	6.37 (1.31)	6.30 (.90)	*t*(345) = .32, *p* = .75, *d* = −.06
PBC capacity	6.27 (1.4)	6.56 (.71)	*t*(345) = −1.68, *p* = .09, *d* = .24
General beliefs	6.27 (1.34)	6.59 (.68)	*t*(345) = −1.94, *p* = .05, *d* = .28

**Table 3:** Bivariate correlation analysis for wearing a mask.

	M (SD)	1	2	3	4	5	6	7	8	9
1. Wearing a mask	4.65 (.89)	–								
2. Intention	6.77 (.61)	.13*	–							
3. Instrumental Attitude	6.22 (1.20)	.03	.37**	–						
4. Experiential Attitude	6.03 (.95)	.07	.46**	.73**	–					
5. Injunctive Norms	6.29 (.79)	.10	.36**	.36**	.38**	–				
6. Descriptive Norms	6.14 (.84)	.14**	.26**	.23**	.23**	.60**	–			
7. PBC Autonomy	6.31 (.92)	.07	.46**	.36**	.44**	.54**	.43**	–		
8. PBC Capacity	6.54 (.77)	.15**	.56**	.48**	.52**	.58**	.46**	.74**	–	
9. General beliefs	6.56 (.75)	.10	.56**	.37**	.47**	.23**	.15**	.39**	.46**	–

Note: *p < .05, ** *p* < .01.

B. Hand washing

The measurement of determinants related to hand washing were grouped into experiential attitude (11 items, α = 0.81, e.g., “Washing my hands with soap or hand sanitizer after touching objects, before touching my face or eating, and before entering the house after traveling during the pandemic makes me feel healthier”), instrumental attitude (9 items, α = 0.97, e.g., “Washing hands with soap or hand sanitizer after touching objects, before touching the face or eating, and before entering the house after traveling during the pandemic reduces the chances of my family transmitting the Covid-19 virus to me”), descriptive norms (14 items, α = 0.93, e.g., “My parents wash their hands with soap or hand sanitizer after touching objects, before touching the face or eating, and before entering the house after traveling during the pandemic”), injunctive norms (14 items, α = 0.93, e.g., “My parents expect me to always wash my hands with soap or hand sanitizer after touching objects, before touching my face or eating, and before entering the house after traveling during the pandemic”), perceived capacity (13 items, α = 0.96, e.g., “I believe I can wash my hands with soap or hand sanitizer after touching objects, before touching the face or eating while doing daily activities (e.g. work, school) during the pandemic”), perceived autonomy (11 items, α = 0.97, e.g., “How far you can control yourself to wash your hands with soap or hand sanitizer after touching objects, before touching your face or eating while doing daily activities outdoors (e.g., work, school) during the pandemic”), and intentions (4 items, α = 0.82, e.g., “I intend to wash my hands with soap or hand sanitizer after touching objects, before touching my face or eating and before entering the house after traveling during the pandemic even though it requires additional costs (to buy soap/hand sanitizer)”). Most of the participants (87,9%) reported washing their hands more frequently during the past week. Detailed information about the socio-demographic difference among participants who showed less vs. more frequent hand washing and psychosocial determinants' mean difference are shown in Tables 4 and 5, respectively. The correlations among study variables related to hand washing were reported in Table 6.

**Table 4:** Sociodemographic characteristics of the participants for hand washing.

	Less frequent		More frequent		
	N	%	N	%	Statistics
**Age**					*χ^2^*(1) = 1.71, *p* = .19, *V* = .08
18–30	31	10.4	201	67.2	
31–65	5	1.7	62	20.7	
**Education**					*χ^2^*(1) = .11, *p* = .74, *V* = .02
Lower education (up to senior high school)	14	4.7	110	36.8	
Higher Education (university)	22	7.4	153	51.2	
**Employment**					*χ^2^*(2) = 2.21, *p* = .33, *V* = .09
Unemployment, student, WFH	24	8	203	67.9	
WFO	2	.7	7	2.3	
Health professional	10	3.3	53	17.8	
**Family monthly income**					*χ^2^*(1) = 3.68, *p* = .06, *V* = .11
Below national standard	22	7.4	116	38.8	
National standard and above	14	4.7	147	49.2	
**History of Covid-19**					*χ^2^*(1) = 4.97, *p* = .03*, *V* = .13
Yes	13	4.3	52	17.4	
No	23	7.7	211	70.6	

Note: *p < .05.

**Table 5:** Comparison of RAA determinants and general beliefs for hand washing.

	Less frequent	More frequent	Statistics
	Mean (SD)	Mean (SD)	
Intention	5.84 (.95)	6.61 (.55)	*t*(297) = −7.08, *p* < .01**, *d* = 1.28
Instrumental attitude	5.83 (.92)	6.21 (1.04)	*t*(297) = −2.09, *p* = .04*, *d* = .39
Experiential attitude	5.75 (.69)	6.08 (.65)	*t*(297) = −2.84, *p* < .01**, *d* *=* *.*73
Injunctive Norms	5.39 (.95)	6.15 (.67)	*t*(297) = −5.97, *p* < .01**, *d* = 1.12
Descriptive Norms	5.34 (1.08)	5.98 (.69)	*t*(297) = −4.84, *p* < .01**, *d* = .78
PBC autonomy	5.32 (1.32)	6.37 (.67)	*t*(297) = −7.57, *p* < .01**, *d* = .96
PBC capacity	5.81 (.93)	6.49 (.59)	*t*(297) = −5.90, *p* < .01**, *d* = 1.12
General beliefs	6.15 (1.08)	6.66 (.74)	*t*(297) = −3.66, *p* < .01**, *d* = .60

Note: *p < .05, ** *p* < .01.

**Table 6:** Bivariate correlation analysis for hand washing.

	M (SD)	1	2	3	4	5	6	7	8	9
1. Hand washing	4.08 (1.07)	–								
2. Intention	6.52 (.66)	.46*	–							
3. Instrumental Attitude	6.16 (1.03)	.26*	.30*	–						
4. Experiential Attitude	6.04 (.67)	.25*	.30*	.74*	–					
5. Injunctive Norms	6.05 (.75)	.35*	.32*	.31*	.27*	–				
6. Descriptive Norms	5.91 (.77)	.34*	.28*	.41*	.34*	.75*	–			
7. PBC Autonomy	6.24 (.85)	.43*	.56*	.23*	.31*	.50*	.43*	–		
8. PBC Capacity	6.41 (.68)	.39*	.51*	.29*	.35*	.44*	.45*	.76*	–	
9. General beliefs	6.60 (.80)	.29*	.19*	.17*	.18*	.15*	.22*	.20*	.23*	–

Note: * *p* < 0.01.

C. Distancing

The measurement of determinants related to distancing were grouped into experiential attitude (13 items, α = 0.89, e.g., “Keeping at least 1 m distance in crowded places during the pandemic makes me feel weird”), instrumental attitude (12 items, α = 0.96, e.g., “Keeping at least 1 m distance in crowded places during the pandemic reduces the chances of others transmitting the Covid-19 virus to me”), descriptive norms (15 items, α = 0.90, e.g., “My colleagues keep at least 1 m distance in crowded places during the pandemic”), injunctive norms (15 items, α = 0.92, e.g., “My colleagues expect me to always keep at least 1 m distance in crowded places during the pandemic”), perceived capacity (14 items, α = 0.94, e.g., “I believe I can keep at least 1 m distance when meeting my family during the pandemic”), perceived autonomy (14 items, α = 0.94, e.g., “How far you can control yourself to keep at least 1 m distance when you meet your family during the pandemic”), and intentions (4 items, α = 0.81, e.g., “I intend to keep a distance 1 m in crowded places during the pandemic”). Most of the participants (78.8%) reported distancing more frequently during the past week. Detailed information about the socio-demographic difference among participants who less vs. more frequently distancing and psychosocial determinants' mean difference are shown in Tables 7 and 8, respectively. The correlations among study variables related to distancing were reported in Table 9.

**Table 7:** Sociodemographic characteristics of the participants for distancing.

	Less frequent		More frequent		
	N	%	N	%	Statistics
**Age**					*χ^2^*(1) = 3.03, *p* = .08, *V* = .12
18–30	40	18.4	128	59	
31–65	6	2.8	43	19.8	
**Education**					*χ^2^*(1) = .31, *p* = .58, *V* = .04
Lower education (up to senior high school)	19	8.8	63	29	
Higher Education (university)	27	12.4	108	49.8	
**Employment**					*χ^2^*(2) = .32, *p* = .85, *V* = .04
Unemployment, student, WFH	33	15.2	125	57.6	
WFO	12	5.5	40	18.4	
Health professional	1	.5	6	2.8	
**Family monthly income**					*χ^2^*(1) = 1.95, *p* = .16, *V* = .10
Below national standard	29	13.4	88	40.6	
National standard and above	17	7.8	83	38.2	
**History of Covid-19**					*χ^2^*(1) = .01, *p* = .92, *V* = .01
Yes	7	3.2	25	11.5	
No	39	18	146	67.3

**Table 8:** Comparison of RAA determinants and general beliefs for distancing.

	Less frequent	More frequent	Statistics
	Mean (SD)	Mean (SD)	
Intention	5.74 (1.07)	6.47 (.59)	*t*(215) = −6.05, *p* < .01*, *d* = .98
Instrumental attitude	5.63 (1.40)	6.12 (.98)	*t*(215) = −2.70, *p* < .01*, *d* = .34
Experiential attitude	5.14 (1.02)	5.95 (.83)	*t*(215) = −5.57, *p* < .01*, *d* = .94
Injunctive Norms	5.11 (1.14)	5.70 (.82)	*t*(215) = −3.95, *p* < .01*, *d* = .60
Descriptive Norms	4.93 (1.24)	5.49 (.83)	*t*(215) = −3.64, *p* < .01*, *d* = .50
PBC autonomy	4.77 (1.32)	5.66 (.84)	*t*(215) = −5,56, *p* < .01*, *d* = .73
PBC capacity	4.85 (1.32)	5.85 (.81)	*t(*215) = −6.29, *p* < .01*, *d* = .83
General beliefs	6.25 (1.21)	6.60 (.63)	*t*(219) = −2.66, *p* < .01*, *d* = .38

Note: *p < .01.

**Table 9:** Bivariate correlation analysis for distancing.

	M (SD)	1	2	3	4	5	6	7	8	9
1. Distancing	3.61 (1.33)	–								
2. Intention	6.32 (.77)	.45**	–							
3. Instrumental Attitude	6.01 (1.10)	.17*	.25**	–						
4. Experiential Attitude	5.77 (.94)	.37**	.42**	.64**	–					
5. Injunctive Norms	5.59 (.93)	.25**	.25**	.45**	.24**	–				
6. Descriptive Norms	5.38 (.96)	.23**	.28**	.45**	.34**	.60**	–			
7. PBC Autonomy	5.48 (1.03)	.38**	.44**	.31**	.31**	.47**	.49**	–		
8. PBC Capacity	5.64 (1.04)	.40**	.48**	.28**	.33**	.50**	.54**	.82**	–	
9. General beliefs	6.53 (.81)	.16*	.16*	.26**	.31**	.08	.10	.12	.11	–

Note: *p < .05, ** *p* < .01.

## 2 Experimental Design, Materials and Methods

The research adopted a cross-sectional survey design to explore the determinants of Covid-19 preventive behaviors, containing three sets of questionnaires related to wearing a mask, hand washing, and physical distancing, where the potential participants may opt to fill in a minimum one of them. The online survey was collected from 18th June until 18th August 2021, with a total of 1002 responses (i.e., wearing a mask: 413 responses, hand washing: 329 responses, and distancing 260 responses) from all provinces in Indonesia. Only cases with completed responses (*n* = 863, accounted for 86.5% from total responses) were included in the final analysis. The questionnaires were designed and distributed using Google Forms and links were shared on social media, i.e., WhatsApp, Instagram, Twitter, and Facebook. Once a participant clicked the link to this study, (s)he was presented with a page containing three links to the scales based on this list order: wearing a mask, hand washing, and distancing, with an instruction that they need to complete a minimum one set of them (i.e., a participant can complete one set of wearing a mask scale or hand washing scale or distancing scale only without complete the other two scales). However, there was a possibility the three datasets containing the same participants among them, since some participants may complete more than one measurement. Additionally, this study used a combination of purposive and snowball techniques to collect the data. The inclusion criteria were (1) Indonesian who was currently living in Indonesia and (2) being 18 years or older. Ethical approval was obtained from the Faculty of Nursing Ethical Board, University of Airlangga. Respondents’ participation was anonymous and voluntary, and all the respondents provided written informed consent prior to participation.

We constructed three sets of questionnaires measuring the beliefs related to Covid-19 preventive behaviors according to the RAA framework by following the recommendation of Ajzen [3]: The questionnaires were generated from a preliminary survey that used open-ended questions to identify specific determinants of Covid-19 prevention behaviors relevant to the Indonesian context, which form direct measures of attitudes (instrumental attitudes vs. experiential attitudes), subjective norms (descriptive norms vs. injunctive norms), and perceived behavioral controls (perceived capacity vs. perceived autonomy). The original questionnaires of beliefs related to wearing a mask, hand washing, and distancing consisted of 88, 85, and 90 items, respectively. Further, we deleted some items based on factor and reliability analyses: An item with communality less than 0.4 in the factor analysis and a corrected item-total correlation less than 0.3 in the reliability test was omitted and not included in the final analysis. Therefore, there were 80, 76, and 87 items of beliefs related to wearing a mask, hand washing, and distancing, respectively, included in the final analysis. Additionally, general beliefs about Covid-19 [4], Covid-19 prevention behaviors, and socio-demographic information were also measured.

Data were analyzed using IBM SPSS. The unfavorable items were reverse-coded. Further, the final items (i.e., after factor and reliability analysis, and reverse-coded) of RAA constructs and general beliefs of Covid-19 for each participant were averaged into a single measure to represent each psychosocial variable. The socio-demographic characteristics of the respondents were analyzed using frequency distributions. Furthermore, bivariate correlation analysis was employed to measure the univariate association between study variables.

## Ethics Statements

Ethical approval was obtained from the Health Research Ethics Commission, Faculty of Nursing, Universitas Airlangga. The protocol number of ethical approvals is 2208-KEPK. Respondents’ participation was completely anonymous and voluntary, and all the respondents provided written informed consent prior to participation.

## CRediT authorship contribution statement

**Triana Kesuma Dewi:** Conceptualization, Methodology, Formal analysis, Visualization, Writing – original draft. **Dyah Ayu Savitri:** Investigation, Formal analysis, Visualization, Writing – original draft. **Muchlisah Audina Sudirman:** Investigation, Formal analysis, Visualization. **Ratri Aisyah Rohmatullaili:** Investigation, Formal analysis, Visualization. **Shafira Rahmadianti:** Investigation, Formal analysis, Visualization. **Rahkman Ardi:** Funding acquisition, Formal analysis, Writing – review & editing.

## Declaration of Competing Interest

The authors declare that they have no known competing financial interests or personal relationships that could have appeared to influence the work reported in this paper.

## Data Availability

Covid-19 preventive behaviors during the second waves in Indonesia using the reasoned action approach (Original data) (OSF).

## Appendix Supplementary materials

Image, application 1
