# Supplementary material for: Survey data of determinants related to Covid-19 preventive behaviors during the second waves in Indonesia using the reasoned action approach
Source: Data Brief. 2022 Apr 9;42:108147. doi: 10.1016/j.dib.2022.108147 (PMC8993698; doi:10.1016/j.dib.2022.108147)
Supplement: Supplementary file 1 [file mmc1.docx]

**QUESTIONNAIRES**

**DETERMINANTS OF COVID-19 PREVENTIVE BEHAVIORS**

1. **WEARING MASK**

|  | **No. Question** | **English Version** | **Bottom Anchor (1)** | **Top Anchor (7)** |
| --- | --- | --- | --- | --- |
| ***Experiential attitude*** | | | | |
| AttExSafe | Q1 | Wearing a mask when leaving the house during the pandemic makes me feel safer | Strongly disagree | Strongly agree |
| AttExHealthy | Q2 | Wearing a mask when leaving the house during the pandemic makes me feel healthier | Strongly disagree | Strongly agree |
| AttExSocRes | Q3 | Wearing a mask when leaving the house during the pandemic makes me feel like I have done my social responsibility to prevent the transmission of Covid-19 | Strongly disagree | Strongly agree |
| AttnExCom | Q4 | Wearing a mask when leaving the house during the pandemic still allows me to communicate with others | Strongly disagree | Strongly agree |
| AttExSilly | Q5 | Wearing a mask when leaving the house during the pandemic makes me feel silly* | Strongly disagree | Strongly agree |
| AttExWeird | Q6 | Wearing a mask when leaving the house during the pandemic makes me feel weird* | Strongly disagree | Strongly agree |
| AttExGood | Q7 | Wearing a mask when leaving the house during the pandemic is a good thing | Strongly disagree | Strongly agree |
| AttExTrouble | Q8 | Wearing a mask when leaving the house during the pandemic is a hassle* | Strongly disagree | Strongly agree |
| AttExSatisfy | Q9 | Wearing a mask when leaving the house during the pandemic makes me feel satisfied with myself | Strongly disagree | Strongly agree |
| AttExComfort | Q10 | Wearing a mask when leaving the house during the pandemic makes me feel composed | Strongly disagree | Strongly agree |
| AttExBurden | Q11 | Wearing a mask when leaving the house during the pandemic makes me feel burdened* | Strongly disagree | Strongly agree |
| AttExObligation | Q12 | Wearing a mask when leaving the house during the pandemic for me is an obligation | Strongly disagree | Strongly agree |
| AttExPriority | Q13 | Wearing a mask when leaving the house during the pandemic is not my priority* | Strongly disagree | Strongly agree |
| ***Instrumental Attitude*** | | | | |
| AttInSelf | Q1 | Wearing a mask when leaving the house during the pandemic keeps me away from the Covid-19 virus | Strongly disagree | Strongly agree |
| AttInSelf1 | Q2 | Wearing a mask when leaving the house during the pandemic prevents me from getting infected by Covid-19 Virus | Strongly disagree | Strongly agree |
| AttInSelf2 | Q3 | Wearing a mask when leaving the house during the pandemic reduces the chances of me getting exposed by Covid-19 virus | Strongly disagree | Strongly agree |
| AttnInFamily | Q4 | Wearing a mask when leaving the house during the pandemic reduces the chances of my family transmitting the Covid-19 virus to me | Strongly disagree | Strongly agree |
| AttnInFamilyFlip | Q5 | Wearing a mask when leaving the house during the pandemic reduces my chances of passing the Covid-19 virus to my family | Strongly disagree | Strongly agree |
| AttnInFriend | Q6 | Wearing a mask when leaving the house during the pandemic reduces the chances of my friend transmitting the Covid-19 virus to me | Strongly disagree | Strongly agree |
| AttnInFriendFlip | Q7 | Wearing a mask when leaving the house during the pandemic reduces my chances of passing the Covid-19 virus to my friend | Strongly disagree | Strongly agree |
| AttnInOther | Q8 | Wearing a mask when leaving the house during the pandemic reduces the chances of others transmitting the Covid-19 virus to me | Strongly disagree | Strongly agree |
| AttnInOtherFlip | Q9 | Wearing a mask when leaving the house during the pandemic reduces my chances of passing the Covid-19 virus to others. | Strongly disagree | Strongly agree |
| ***Descriptive Norms*** | | | | |
| NrmDeFam | Q1 | My family members wear a mask when leaving the house during the pandemic | Strongly disagree | Strongly agree |
| NrmDeParent | Q2 | My parents wear a mask when leaving the house during the pandemic | Strongly disagree | Strongly agree |
| NrmDeFriend | Q3 | My close friends wear a mask when leaving the house during the pandemic | Strongly disagree | Strongly agree |
| NrmDeHealthcare | Q4 | Health workers wear a mask when leaving the house during the pandemic | Strongly disagree | Strongly agree |
| NrmDeNeighbour | Q5 | My neighbours wear a mask when leaving the house during the pandemic | Strongly disagree | Strongly agree |
| NrmDeColleague | Q6 | My colleagues wear a mask when leaving the house during the pandemic | Strongly disagree | Strongly agree |
| NrmDeImportantOthr | Q7 | People close to me wear a mask when leaving the house during the pandemic | Strongly disagree | Strongly agree |
| NrmDeImportantOthr1 | Q8 | People who are important to me wear a mask when leaving the house during the pandemic | Strongly disagree | Strongly agree |
| NrmDePeers | Q9 | People at my age wear a mask when leaving the house during the pandemic | Strongly disagree | Strongly agree |
| NrmDePeers1 | Q10 | People like me wear a mask when leaving the house during the pandemic | Strongly disagree | Strongly agree |
| NrmDeOther | Q11 | Other people wear a mask when leaving the house during the pandemic | Strongly disagree | Strongly agree |
| NrmDeGeneral | Q12 | People generally wear a mask when leaving the house during the pandemic | Strongly disagree | Strongly agree |
| NrmDeRoleModel | Q13 | People I admire wear a mask when leaving the house during the pandemic | Strongly disagree | Strongly agree |
| NrmDeBoss | Q14 | My boss wears a mask when leaving the house during the pandemic | Strongly disagree | Strongly agree |
| ***Injunctive Norms*** | | | | |
| NrmInPartner | Q1 | My partner expects me to always wear a mask when leaving the house during the pandemic | Strongly disagree | Strongly agree |
| NrmInFam | Q2 | My family expects me to always wear a mask when leaving the house during the pandemic | Strongly disagree | Strongly agree |
| NrmInParent | Q3 | My parents expect me to always wear a mask when leaving the house during the pandemic | Strongly disagree | Strongly agree |
| NrmInFriend | Q4 | My close friends expect me to always wear a mask when leaving the house during the pandemic | Strongly disagree | Strongly agree |
| NrmInHealthcare | Q5 | Health workers expect me to wear a mask when leaving the house during the pandemic | Strongly disagree | Strongly agree |
| NrmInNeighbour | Q6 | My neighbours expect me to always wear a mask when leaving the house during the pandemic | Strongly disagree | Strongly agree |
| NrmInColleague | Q7 | My colleagues expect me to always wear a mask when leaving the house during the pandemic | Strongly disagree | Strongly agree |
| NrmInGovern | Q8 | The government expects me to always wear a mask when leaving the house during the pandemic | Strongly disagree | Strongly agree |
| NrmInImportantOthr | Q9 | People who are important to me expect me to always wear a mask when leaving the house during the pandemic | Strongly disagree | Strongly agree |
| NrmInPeers1 | Q10 | People at my age expect me to always wear a mask when leaving the house during the pandemic | Strongly disagree | Strongly agree |
| NrmInPeers2 | Q11 | People like me expect me to always wear a mask when leaving the house during the pandemic | Strongly disagree | Strongly agree |
| NrmInOthers | Q12 | Other people expect me to always wear a mask when leaving the house during the pandemic | Strongly disagree | Strongly agree |
| NrmInGeneral | Q13 | People generally expect me to always wear a mask when leaving the house during the pandemic | Strongly disagree | Strongly agree |
| NrmInRoleModel | Q14 | People I admire expect me to always wear a mask when leaving the house during the pandemic | Strongly disagree | Strongly agree |
| NrmInBoss | Q15 | My boss expects me to always wear a mask when leaving the house during the pandemic | Strongly disagree | Strongly agree |
| ***Perceived Capacity*** | | | | |
| PbcCaNeighbour | Q1 | I believe I can wear a mask when I'm around the neighborhood where I lived during the pandemic | Strongly disagree | Strongly agree |
| PbcCaShopping | Q2 | I believe I can wear a mask when shopping at the store/supermarket/market during the pandemic | Strongly disagree | Strongly agree |
| PbcCaSport | Q3 | I believe I can wear a mask when exercising outside the house during the pandemic | Strongly disagree | Strongly agree |
| PbcCaDailyActive | Q4 | I believe I can wear a mask while doing daily activities (e.g. work, school) during the pandemic. | Strongly disagree | Strongly agree |
| PbcCaKnown | Q5 | I believe I can wear a mask when I go to a familiar environment during the pandemic | Strongly disagree | Strongly agree |
| PbcCaFamily | Q6 | I believe I can wear a mask when when meeting my family during the pandemic | Strongly disagree | Strongly agree |
| PbcCaFamily1 | Q7 | I believe I can wear a mask when visiting my family during the pandemic | Strongly disagree | Strongly agree |
| PbcCaFriend | Q8 | I believe I can wear a mask when meeting my friends during the pandemic | Strongly disagree | Strongly agree |
| PbcCaFriend1 | Q9 | I believe I can wear a mask when visiting my friends during the pandemic | Strongly disagree | Strongly agree |
| PbcCaOlder | Q10 | I believe I can wear a mask when communicating with older people during the pandemic | Strongly disagree | Strongly agree |
| PbcCaCleanMask | Q11 | I believe I can provide clean masks to use when I leave the house during the pandemic | Strongly disagree | Strongly agree |
| PbcCaAccess | Q12 | I believe I can provide clean masks in places that are easily accessible whenever needed during the pandemic | Strongly disagree | Strongly agree |
| ***Perceived autonomy*** | | | | |
| PbcAuNeighbour | Q1 | How far you can control yourself to keep wearing a mask when you are in your neighborhood during the pandemic | Very unlikely can control | Very likely can control |
| PbcAuShopping | Q2 | How far you can control yourself to keep wearing a mask when shopping at the store/supermarket/market during the pandemic | Very unlikely can control | Very likely can control |
| PbcAuSport | Q3 | How far you can control yourself to keep wearing a mask when when exercising outside during the pandemic | Very unlikely can control | Very likely can control |
| PbcAuDailyActive | Q4 | How far you can control yourself to keep wearing a mask while doing daily activities outdoors (e.g. work, school) during the pandemic | Very unlikely can control | Very likely can control |
| PbcAuKnown | Q5 | How far you can control yourself to keep wearing a mask when you go to a familiar environment during the pandemic | Very unlikely can control | Very likely can control |
| PbcAuFamily | Q6 | How far you can control yourself to keep wearing a mask when you meet your family during the pandemic | Very unlikely can control | Very likely can control |
| PbcAuFamily1 | Q7 | How far you can control yourself to keep wearing a mask when visiting your family during the pandemic | Very unlikely can control | Very likely can control |
| PbcAuFriend | Q8 | How far you can control yourself to keep wearing a mask when you meet your friends during the pandemic | Very unlikely can control | Very likely can control |
| PbcAuFriend1 | Q9 | How far you can control yourself to keep wearing a mask when visiting your friends during the pandemic | Very unlikely can control | Very likely can control |
| PbcAuClosePerson | Q10 | How far you can control yourself to keep wearing a mask when you are around people you know closely during the pandemic | Very unlikely can control | Very likely can control |
| PbcAuOlder | Q11 | How far you can control yourself to keep wearing a mask when when you talk to older people during the pandemic | Very unlikely can control | Very likely can control |
| PbcAuAsked | Q12 | How far you can control yourself to keep wearing a mask when other people ask you to take off your mask during a pandemic | Very unlikely can control | Very likely can control |
| PbcAuNoMask | Q13 | How far you can control yourself to keep wearing a mask when you are in an environment that is mostly not wearing a mask during the pandemic | Very unlikely can control | Very likely can control |
| ***Intention*** | | | | |
| IntMask | Q1 | I intend to wear a mask when leaving the house during the pandemic | Strongly disagree | Strongly agree |
| IntInRush | Q2 | I intend to wear a mask even though I'm in a hurry to leave the house during the pandemic | Strongly disagree | Strongly agree |
| IntTrouble | Q3 | I intend to wear a mask when leaving the house during the pandemic even though it's troublesome | Strongly disagree | Strongly agree |
| IntCost | Q4 | I intend to wear a mask when leaving the house during the pandemic even though it requires additional costs (to buy a mask) | Strongly disagree | Strongly agree |
| ***General beliefs*** | | | | |
| GenBelReal | Q1 | Corona Virus and Covid-19 | Hoax | Real |
| GenBelIndoor | Q2 | Getting infected with corona virus by other people | Can only occur in a closed room (indoor) | Can occur in an open space (outdoor) or a closed room (indoor) |
| GenBelSymptomatic | Q3 | Getting infected with corona virus by other people | Can only occur when the person has symptoms | Can occur even though the person is asymptomatic |

Note: * unfavorable item

1. **WASHING HANDS**

|  | **No. Question** | **English Version** | **Bottom Anchor (1)** | **Top Anchor (7)** |
| --- | --- | --- | --- | --- |
| ***Experiential attitude*** | | | | |
| AttExSafe | Q1 | Washing my hands with soap or hand sanitizer after touching objects, before touching my face or eating, and before entering the house after traveling during the pandemic makes me feel safer | Strongly disagree | Strongly agree |
| AttExHealthy | Q2 | Washing my hands with soap or hand sanitizer after touching objects, before touching my face or eating, and before entering the house after traveling during the pandemic makes me feel healthier | Strongly disagree | Strongly agree |
| AttExSocRes | Q3 | Washing hands with soap or hand sanitizer after touching objects, before touching my face or eating, and before entering the house after traveling during the pandemic makes me feel like I have done my social responsibility to prevent the transmission of Covid-19 | Strongly disagree | Strongly agree |
| AttExSilly | Q4 | Washing my hands with soap or hand sanitizer after touching objects, before touching my face or eating, and before entering the house after traveling during the pandemic makes me feel silly* | Strongly disagree | Strongly agree |
| AttExWeird | Q5 | Washing my hands with soap or hand sanitizer after touching objects, before touching my face or eating, and before entering the house after traveling during the pandemic makes me feel weird* | Strongly disagree | Strongly agree |
| AttExGood | Q6 | Washing hands with soap or hand sanitizer after touching objects, before touching my face or eating, and before entering the house after traveling during the pandemic is a good thing | Strongly disagree | Strongly agree |
| AttExTrouble | Q7 | Washing hands with soap or hand sanitizer after touching objects, before touching my face or eating, and before entering the house after traveling during the pandemic is a hassle* | Strongly disagree | Strongly agree |
| AttExSatisfy | Q8 | Washing my hands with soap or hand sanitizer after touching objects, before touching my face or eating, and before entering the house after traveling during the pandemic makes me feel satisfied with myself | Strongly disagree | Strongly agree |
| AttExComfort | Q9 | Washing my hands with soap or hand sanitizer after touching objects, before touching my face or eating, and before entering the house after traveling during the pandemic makes me feel composed | Strongly disagree | Strongly agree |
| AttExObligation | Q10 | Washing hands with soap or hand sanitizer after touching objects, before touching my face or eating, and before entering the house after traveling during the pandemic for me is an obligation | Strongly disagree | Strongly agree |
| AttExPriority | Q11 | Washing my hands with soap or hand sanitizer after touching objects, before touching my face or eating, and before entering the house after traveling during the pandemic is not my priority* | Strongly disagree | Strongly agree |
| ***Instrumental Attitude*** | | | | |
| AttInSelf | Q1 | Washing my hands with soap or hand sanitizer after touching objects, before touching my face or eating, and before entering the house after traveling during the pandemic keeps me away from the Covid-19 virus | Strongly disagree | Strongly agree |
| AttInSelf1 | Q2 | Washing my hands with soap or hand sanitizer after touching objects, before touching my face or eating, and before entering the house after traveling during the pandemic prevents me from getting infected Covid-19 | Strongly disagree | Strongly agree |
| AttInSelf2 | Q3 | Washing my hands with soap or hand sanitizer after touching objects, before touching my face or eating, and before entering the house after traveling during the pandemic reduces the chances of me getting exposed by Covid-19 virus | Strongly disagree | Strongly agree |
| AttnInFamily | Q4 | Washing hands with soap or hand sanitizer after touching objects, before touching my face or eating, and before entering the house after traveling during the pandemic reduces the chances of my family transmitting the Covid-19 virus to me | Strongly disagree | Strongly agree |
| AttnInFamilyFlip | Q5 | Washing my hands with soap or hand sanitizer after touching objects, before touching my face or eating, and before entering the house after traveling during the pandemic reduces my chances of passing the Covid-19 virus to my family | Strongly disagree | Strongly agree |
| AttnInFriend | Q6 | Washing hands with soap or hand sanitizer after touching objects, before touching my face or eating, and before entering the house after traveling during the pandemic reduces the the chances of my friend transmitting the Covid-19 virus to me | Strongly disagree | Strongly agree |
| AttnInFriendFlip | Q7 | Washing my hands with soap or hand sanitizer after touching objects, before touching my face or eating, and before entering the house after traveling during the pandemic reduces my chances of passing the Covid-19 virus to my friend | Strongly disagree | Strongly agree |
| AttnInOther | Q8 | Washing hands with soap or hand sanitizer after touching objects, before touching my face or eating, and before entering the house after traveling during the pandemic reduces the chances of others transmitting the Covid-19 virus to me | Strongly disagree | Strongly agree |
| AttnInOtherFlip | Q9 | Washing my hands with soap or hand sanitizer after touching objects, before touching my face or eating, and before entering the house after traveling during the pandemic reduces my chances of passing the Covid-19 virus to others | Strongly disagree | Strongly agree |
| ***Descriptive Norms*** | | | | |
| NrmDeFam | Q1 | My family members wash their hands with soap or hand sanitizer after touching objects, before touching my face or eating, and before entering the house after traveling during the pandemic | Strongly disagree | Strongly agree |
| NrmDeParent | Q2 | My parents wash their hands with soap or hand sanitizer after touching objects, before touching my face or eating, and before entering the house after traveling during the pandemic | Strongly disagree | Strongly agree |
| NrmDeFriend | Q3 | My close friends wash their hands with soap or hand sanitizer after touching objects, before touching my face or eating, and before entering the house after traveling during the pandemic | Strongly disagree | Strongly agree |
| NrmDeHealthcare | Q4 | Health workers wash their hands with soap or hand sanitizer after touching objects, before touching my face or eating, and before entering the house after traveling during the pandemic | Strongly disagree | Strongly agree |
| NrmDeNeighbour | Q5 | My neighbours wash their hands with soap or hand sanitizer after touching objects, before touching my face or eating, and before entering the house after traveling during the pandemic | Strongly disagree | Strongly agree |
| NrmDeColleague | Q6 | My colleagues wash their hands with soap or hand sanitizer after touching objects, before touching my face or eating, and before entering the house after traveling during the pandemic | Strongly disagree | Strongly agree |
| NrmDeImportantOthr | Q7 | People close to me wash their hands with soap or hand sanitizer after touching objects, before touching my face or eating, and before entering the house after traveling during the pandemic | Strongly disagree | Strongly agree |
| NrmDeImportantOthr1 | Q8 | People who are important to me wash their hands with soap or hand sanitizer after touching objects, before touching my face or eating, and before entering the house after traveling during the pandemic | Strongly disagree | Strongly agree |
| NrmDePeers | Q9 | People at my age wash their hands with soap or hand sanitizer after touching objects, before touching my face or eating, and before entering the house after traveling during the pandemic | Strongly disagree | Strongly agree |
| NrmDePeers1 | Q10 | People like me washes their hands with soap or hand sanitizer after touching objects, before touching my face or eating, and before entering the house after traveling during the pandemic | Strongly disagree | Strongly agree |
| NrmDeOther | Q11 | Other people wash their hands with soap or hand sanitizer after touching objects, before touching my face or eating, and before entering the house after traveling during the pandemic | Strongly disagree | Strongly agree |
| NrmDeGeneral | Q12 | People generally wash their hands with soap or hand sanitizer after touching objects, before touching my face or eating, and before entering the house after traveling during the pandemic | Strongly disagree | Strongly agree |
| NrmDeRoleModel | Q13 | People I admire wash their hands with soap or hand sanitizer after touching objects, before touching my face or eating, and before entering the house after traveling during the pandemic | Strongly disagree | Strongly agree |
| NrmDeBoss | Q14 | My boss washes my hands with soap or hand sanitizer after touching objects, before touching my face or eating, and before entering the house after traveling during the pandemic | Strongly disagree | Strongly agree |
| ***Injunctive Norms*** | | | | |
| NrmInFam | Q1 | My family expects me to always wash my hands with soap or hand sanitizer after touching objects, before touching my face or eating, and before entering the house after traveling during the pandemic | Strongly disagree | Strongly agree |
| NrmInParent | Q2 | My parents expect me to always wash my hands with soap or hand sanitizer after touching objects, before touching my face or eating, and before entering the house after traveling during the pandemic | Strongly disagree | Strongly agree |
| NrmInFriend | Q3 | My close friends expect me to always wash my hands with soap or hand sanitizer after touching objects, before touching my face or eating, and before entering the house after traveling during the pandemic | Strongly disagree | Strongly agree |
| NrmInHealthcare | Q4 | Health workers expect me to always wash my hands with soap or hand sanitizer after touching objects, before touching my face or eating, and before entering the house after traveling during the pandemic | Strongly disagree | Strongly agree |
| NrmInNeighbour | Q5 | My neighbours expect me to always wash my hands with soap or hand sanitizer after touching objects, before touching the face or eating, and before entering the house after traveling during the pandemic | Strongly disagree | Strongly agree |
| NrmInColleague | Q6 | My colleagues expect me to always wash my hands with soap or hand sanitizer after touching objects, before touching my face or eating, and before entering the house after traveling during the pandemic | Strongly disagree | Strongly agree |
| NrmInGovern | Q7 | The government expects me to always wash my hands with soap or hand sanitizer after touching objects, before touching my face or eating, and before entering the house after traveling during the pandemic | Strongly disagree | Strongly agree |
| NrmInImportantOthr | Q8 | People who are important to me expect me to always wash my hands with soap or hand sanitizer after touching objects, before touching my face or eating, and before entering the house after traveling during the pandemic. | Strongly disagree | Strongly agree |
| NrmInPeers1 | Q9 | People at my age expect me to always wash my hands with soap or hand sanitizer after touching objects, before touching my face or eating, and before entering the house after traveling during the pandemic | Strongly disagree | Strongly agree |
| NrmInPeers2 | Q10 | People like me expect me to always wash my hands with soap or hand sanitizer after touching objects, before touching my face or eating, and before entering the house after traveling during the pandemic | Strongly disagree | Strongly agree |
| NrmInOthers | Q11 | Other people expect me to always wash my hands with soap or hand sanitizer after touching objects, before touching my face or eating, and before entering the house after traveling during the pandemic | Strongly disagree | Strongly agree |
| NrmInGeneral | Q12 | People generally expect me to always wash my hands with soap or hand sanitizer after touching objects, before touching my face or eating, and before entering the house after traveling during the pandemic | Strongly disagree | Strongly agree |
| NrmInRoleModel | Q13 | People I admire expect me to always wash my hands with soap or hand sanitizer after touching objects, before touching my face or eating, and before entering the house after traveling during the pandemic | Strongly disagree | Strongly agree |
| NrmInBoss | Q14 | My boss expects me to always wash my hands with soap or hand sanitizer after touching objects, before touching my face or eating, and before entering the house after traveling during the pandemic | Strongly disagree | Strongly agree |
| ***Perceived Capacity*** | | | | |
| PbcCaNeighbour | Q1 | I believe I can wash my hands with soap or hand sanitizer after touching objects, before touching my face or eating when I'm around the neighborhood where I lived during the pandemic | Strongly disagree | Strongly agree |
| PbcCaShopping | Q2 | I believe I can wash my hands with soap or hand sanitizer after touching objects, before touching my face or eating when shopping at the store/supermarket/market during the pandemic | Strongly disagree | Strongly agree |
| PbcCaSport | Q3 | I believe I can wash my hands with soap or hand sanitizer after touching objects, before touching my face or eating when exercising outside the house during the pandemic | Strongly disagree | Strongly agree |
| PbcCaDailyActive | Q4 | I believe I can wash my hands with soap or hand sanitizer after touching objects, before touching my face or eating while doing daily activities (e.g. work, school) during the pandemic | Strongly disagree | Strongly agree |
| PbcCaKnown | Q5 | I believe I can wash my hands with soap or hand sanitizer after touching objects, before touching my face or eating when I go to a familiar environment during the pandemic | Strongly disagree | Strongly agree |
| PbcCaFamily | Q6 | I believe I can wash my hands with soap or hand sanitizer after touching objects, before touching my face or eating when meeting my family during the pandemic | Strongly disagree | Strongly agree |
| PbcCaFamily1 | Q7 | I believe I can wash my hands with soap or hand sanitizer after touching objects, before touching my face or eating when visiting my family during the pandemic | Strongly disagree | Strongly agree |
| PbcCaFriend | Q8 | I believe I can wash my hands with soap or hand sanitizer after touching objects, before touching my face or eating when meeting my friends during the pandemic | Strongly disagree | Strongly agree |
| PbcCaFriend1 | Q9 | I believe I can wash my hands with soap or hand sanitizer after touching objects, before touching my face or eating when visiting my friend during the pandemic | Strongly disagree | Strongly agree |
| PbcCaClosePerson | Q10 | I believe I can wash my hands with soap or hand sanitizer after touching objects, before touching my face or eating when I'm around people I know closely during the pandemic | Strongly disagree | Strongly agree |
| PbcCaInRush | Q11 | I believe I can wash my hands with soap or hand sanitizer after touching objects, before touching my face or eating and before entering the house after traveling during the pandemic despite being in a hurry | Strongly disagree | Strongly agree |
| PbcCaAvailability | Q12 | I believe I can provide soap and running water or a hand sanitizer that I can use before entering the house after traveling during the pandemic | Strongly disagree | Strongly agree |
| PbcCaAccess1 | Q13 | I am confident that I am able to wash my hands with soap or hand sanitizer after touching objects, before touching my face or eating and before entering the house after traveling during the pandemic although I need to find a hand sanitizer that is quite far from where I am | Strongly disagree | Strongly agree |
| ***Perceived autonomy*** | | | | |
| PbcAuNeighbour | Q1 | How far you can control yourself to wash your hands with soap or hand sanitizer after touching objects, before touching your face or eating when you are in your neighborhood during the pandemic | Very unlikely can control | Very likely can control |
| PbcAuShopping | Q2 | How far you can control yourself to wash your hands with soap or hand sanitizer after touching objects, before touching your face or eating when shopping at the store/supermarket/market during the pandemic | Very unlikely can control | Very likely can control |
| PbcAuSport | Q3 | How far you can control yourself to wash your hands with soap or hand sanitizer after touching objects, before touching your face or eating when exercising outside the house during the pandemic | Very unlikely can control | Very likely can control |
| PbcAuDailyActive | Q4 | How far you can control yourself to wash your hands with soap or hand sanitizer after touching objects, before touching your face or eating while doing daily activities outdoors (e.g. work, school) during the pandemic | Very unlikely can control | Very likely can control |
| PbcAuKnown | Q5 | How far you can control yourself to wash your hands with soap or hand sanitizer after touching objects, before touching your face or eating when you go to a familiar environment during the pandemic | Very unlikely can control | Very likely can control |
| PbcAuFamily | Q6 | How far you can control yourself to wash your hands with soap or hand sanitizer after touching objects, before touching your face or eating when you meet your family during the pandemic | Very unlikely can control | Very likely can control |
| PbcAuFamily1 | Q7 | How far you can control yourself to wash your hands with soap or hand sanitizer after touching objects, before touching your face or eating when visiting your family during the pandemic | Very unlikely can control | Very likely can control |
| PbcAuFriend | Q8 | How far you can control yourself to wash your hands with soap or hand sanitizer after touching objects, before touching your face or eating when you meet your friends during the pandemic | Very unlikely can control | Very likely can control |
| PbcAuFriend1 | Q9 | How far you can control yourself to wash your hands with soap or hand sanitizer after touching objects, before touching your face or eating when visiting your friends during the pandemic | Very unlikely can control | Very likely can control |
| PbcAuClosePerson | Q10 | How far you can control yourself to wash your hands with soap or hand sanitizer after touching objects, before touching your face or eating when you are around people you know closely during the pandemic | Very unlikely can control | Very likely can control |
| PbcAuOlder | Q11 | How far you can control yourself to wash your hands with soap or hand sanitizer after touching objects, before touching your face or eating when you talk to older people during the pandemic | Very unlikely can control | Very likely can control |
| ***Intention*** | | | | |
| IntWashHand | Q1 | I intend to wash my hands with soap or hand sanitizer after touching objects, before touching my face or eating and before entering the house after traveling during the pandemic | Strongly disagree | Strongly agree |
| IntInRush | Q2 | I intend to wash my hands with soap or hand sanitizer after touching objects, before touching my face or eating and before entering the house after traveling during the pandemic despite being in a hurry | Strongly disagree | Strongly agree |
| IntTrouble | Q3 | I intend to wash my hands with soap or hand sanitizer after touching objects, before touching my face or eating and before entering the house after traveling during the pandemic even though it is troublesome | Strongly disagree | Strongly agree |
| IntCost | Q4 | I intend to wash my hands with soap or hand sanitizer after touching objects, before touching my face or eating and entered the house after traveling during the pandemic even though it requires additional costs (to buy soap/hand sanitizer) | Strongly disagree | Strongly agree |
| ***General beliefs*** | | | | |
| GenBelReal | Q1 | Corona Virus and Covid-19 | Hoax | Real |
| GenBelSymptomatic | Q2 | Getting infected with corona virus by other people | Can only occur when the person has symptoms | Can occur even though the person is asymptomatic |

Note: * unfavorable item

1. **DISTANCING**

|  | **No. Question** | **English Version** | **Bottom Anchor (1)** | **Top Anchor (7)** |
| --- | --- | --- | --- | --- |
| ***Experiential attitude*** | | | | |
| AttExSafe | Q1 | Keeping at least 1 meter distance in crowded places during the pandemic makes me feel safer | Strongly disagree | Strongly agree |
| AttExHealthy | Q2 | Keeping at least 1 meter distance in crowded places during the pandemic makes me feel healthier | Strongly disagree | Strongly agree |
| AttExSocRes | Q3 | Keeping at least 1 meter distance in crowded places during pandemic makes me feel like I have done my social responsibility to prevent the transmission of Covid-19 | Strongly disagree | Strongly agree |
| AttnExCom | Q4 | Keeping at least 1 meter distance in crowded places during the pandemic disrupt my interaction with others* | Strongly disagree | Strongly agree |
| AttExSilly | Q5 | Keeping at least 1 meter distance in crowded places during the pandemic makes me feel silly* | Strongly disagree | Strongly agree |
| AttExWeird | Q6 | Keeping at least 1 meter distance in crowded places during the pandemic makes me feel weird* | Strongly disagree | Strongly agree |
| AttExGood | Q7 | Keeping at least 1 meter distance in crowded places during the pandemic is a good thing | Strongly disagree | Strongly agree |
| AttExTrouble | Q8 | Keeping at least 1 meter distance in crowded places during the pandemic is a hassle* | Strongly disagree | Strongly agree |
| AttExSatisfy | Q9 | Keeping at least 1 meter distance in crowded places during the pandemic makes me feel satisfied with myself | Strongly disagree | Strongly agree |
| AttExComfort | Q10 | Keeping at least 1 meter distance in crowded places during the pandemic makes me feel composed | Strongly disagree | Strongly agree |
| AttExBurden | Q11 | Keeping at least 1 meter distance in crowded places during the pandemic makes me feel burdened* | Strongly disagree | Strongly agree |
| AttExObligation | Q12 | Keeping at least 1 meter distance in crowded places during the pandemic for me is an obligation | Strongly disagree | Strongly agree |
| AttExPriority | Q13 | Keeping at least 1 meter distance in crowded places during the pandemic is not my priority* | Strongly disagree | Strongly agree |
| ***Instrumental Attitude*** | | | | |
| AttInRespect | Q1 | Keeping at least 1 meter distance in crowded places during the pandemic makes me feel more appreciated | Strongly disagree | Strongly agree |
| AttInSelf | Q2 | Keeping at least 1 meter distance in crowded places during the pandemic keeps me from the Covid-19 virus | Strongly disagree | Strongly agree |
| AttInSelf1 | Q3 | Keeping at least 1 meter distance in crowded places during the pandemic prevents me from getting infected by Covid-19 | Strongly disagree | Strongly agree |
| AttInSelf2 | Q4 | Keeping at least 1 meter distance in crowded places during the pandemic reduces the chances of me getting exposed by Covid-19 virus | Strongly disagree | Strongly agree |
| AttInSelf3 | Q5 | Keeping at least 1 meter distance in crowded places during the pandemic reduces the chances of me getting infected by Covid-19 virus | Strongly disagree | Strongly agree |
| AttnInFamily | Q6 | Keeping at least 1 meter distance in crowded places during the pandemic reduces the chances of my family transmitting the Covid-19 virus to me | Strongly disagree | Strongly agree |
| AttnInFamilyFlip | Q7 | Keeping at least 1 meter distance in crowded places during the pandemic reduces my chances of passing the Covid-19 virus to my family | Strongly disagree | Strongly agree |
| AttnInFriend | Q8 | Keeping at least 1 meter distance in crowded places during the pandemic reduces the chances of my friend transmitting the Covid-19 virus to me | Strongly disagree | Strongly agree |
| AttnInFriendFlip | Q9 | Keeping at least 1 meter distance in crowded places during the pandemic reduces my chances of passing the Covid-19 virus to my friend | Strongly disagree | Strongly agree |
| AttnInOther | Q10 | Keeping at least 1 meter distance in crowded places during the pandemic reduces the chances of others transmitting the Covid-19 virus to me | Strongly disagree | Strongly agree |
| AttnInOtherFlip | Q11 | Keeping at least 1 meter distance in crowded places during the pandemic reduces my chances of passing the Covid-19 virus to others. | Strongly disagree | Strongly agree |
| AttInComfort | Q12 | Keeping at least 1 meter distance in crowded places during the pandemic makes me feel more comfortable | Strongly disagree | Strongly agree |
| ***Descriptive Norms*** | | | | |
| NrmDePartner | Q1 | My partner keeps 1 meter distance in crowded places during the pandemic | Strongly disagree | Strongly agree |
| NrmDeFam | Q2 | My family members keep at least 1 meter distance in crowded places during the pandemic | Strongly disagree | Strongly agree |
| NrmDeParent | Q3 | My parents keep at least 1 meter distance in crowded places during the pandemic | Strongly disagree | Strongly agree |
| NrmDeFriend | Q4 | My close friends keep at least 1 meter distance in crowded places during the pandemic | Strongly disagree | Strongly agree |
| NrmDeHealthcare | Q5 | Health workers keep at least 1 meter distance in crowded places during the pandemic | Strongly disagree | Strongly agree |
| NrmDeNeighbour | Q6 | My neighbours keep at least 1 meter distance in crowded places during the pandemic | Strongly disagree | Strongly agree |
| NrmDeColleague | Q7 | My colleagues keep at least 1 meter distance in crowded places during the pandemic | Strongly disagree | Strongly agree |
| NrmDeImportantOthr | Q8 | My close people keep at least 1 meter distance in crowded places during the pandemic | Strongly disagree | Strongly agree |
| NrmDeImportantOthr1 | Q9 | People who are important to me keep at least 1 meter distance in crowded places during the pandemic | Strongly disagree | Strongly agree |
| NrmDePeers | Q10 | People at my age keep at least 1 meter distance in crowded places during the pandemic | Strongly disagree | Strongly agree |
| NrmDePeers1 | Q11 | People like me keep at least 1 meter distance in crowded places during the pandemic | Strongly disagree | Strongly agree |
| NrmDeOther | Q12 | Other people keep at least 1 meter distance in crowded places during the pandemic | Strongly disagree | Strongly agree |
| NrmDeGeneral | Q13 | People generally keep at least 1 meter distance in crowded places during the pandemic | Strongly disagree | Strongly agree |
| NrmDeRoleModel | Q14 | People I admire keep at least 1 meter distance in crowded places during the pandemic | Strongly disagree | Strongly agree |
| NrmDeBoss | Q15 | My boss keeps 1 meter distance in crowded places during the pandemic | Strongly disagree | Strongly agree |
| ***Injunctive Norms*** | | | | |
| NrmInPartner | Q1 | My partner expects me to always keep at least 1 meter distance in crowded places during the pandemic | Strongly disagree | Strongly agree |
| NrmInFam | Q2 | My family expects me to always keep at least 1 meter distance in crowded places during the pandemic | Strongly disagree | Strongly agree |
| NrmInParent | Q3 | My parents expect me to always keep at least 1 meter distance in crowded places during the pandemic | Strongly disagree | Strongly agree |
| NrmInFriend | Q4 | My close friends expect me to always keep at least 1 meter distance in crowded places during the pandemic | Strongly disagree | Strongly agree |
| NrmInHealthcare | Q5 | Health workers expect me to always keep at least 1 meter distance in crowded places during the pandemic | Strongly disagree | Strongly agree |
| NrmInNeighbour | Q6 | My neighbours expect me to always keep at least 1 meter distance in crowded places during the pandemic | Strongly disagree | Strongly agree |
| NrmInColleague | Q7 | My colleagues expect me to always keep at least 1 meter distance in crowded places during the pandemic | Strongly disagree | Strongly agree |
| NrmInGovern | Q8 | The government expects me to always keep at least 1 meter distance in crowded places during the pandemic | Strongly disagree | Strongly agree |
| NrmInImportantOthr | Q9 | People who are important to me expect me to always keep at least 1 meter distance in crowded places during the pandemic | Strongly disagree | Strongly agree |
| NrmInPeers1 | Q10 | People at my age expect me to always keep at least 1 meter distance in crowded places during the pandemic | Strongly disagree | Strongly agree |
| NrmInPeers2 | Q11 | People like me expect me to always keep at least 1 meter distance in crowded places during the pandemic | Strongly disagree | Strongly agree |
| NrmInOthers | Q12 | Other people expect me to always keep at least 1 meter distance in crowded places during the pandemic | Strongly disagree | Strongly agree |
| NrmInGeneral | Q13 | People generally expect me to always keep at least 1 meter distance in crowded places during the pandemic | Strongly disagree | Strongly agree |
| NrmInRoleModel | Q14 | People I admire expect me to always keep at least 1 meter distance in crowded places during the pandemic | Strongly disagree | Strongly agree |
| NrmInBoss | Q15 | My boss expects me to always keep at least 1 meter distance in crowded places during the pandemic | Strongly disagree | Strongly agree |
| ***Perceived Capacity*** | | | | |
| PbcCaNeighbour | Q1 | I believe I can keep at least 1 meter distance when I'm around the neighborhood where I lived during the pandemic | Strongly disagree | Strongly agree |
| PbcCaShopping | Q2 | I believe I can keep at least 1 meter distance when shopping at the store/supermarket/market during the pandemic | Strongly disagree | Strongly agree |
| PbcCaSport | Q3 | I believe I can keep at least 1 meter distance when exercising outside the house during the pandemic | Strongly disagree | Strongly agree |
| PbcCaDailyActive | Q4 | I believe I can keep at least 1 meter distance while doing daily activities (e.g. work, school) during the pandemic | Strongly disagree | Strongly agree |
| PbcCaKnown | Q5 | I believe I can keep at least 1 meter distance when I go to familiar places during the pandemic | Strongly disagree | Strongly agree |
| PbcCaFamily | Q6 | I believe I can keep at least 1 meter distance when meeting my family during the pandemic | Strongly disagree | Strongly agree |
| PbcCaFamily1 | Q7 | I believe I can keep at least 1 meter distance when visiting my family during the pandemic | Strongly disagree | Strongly agree |
| PbcCaFriend | Q8 | I believe I can keep at least 1 meter distance when meeting my friends during the pandemic | Strongly disagree | Strongly agree |
| PbcCaFriend1 | Q9 | I believe I can keep at least 1 meter distance when visiting my friends during the pandemic | Strongly disagree | Strongly agree |
| PbcCaClosePerson | Q10 | I believe I can keep at least 1 meter distance when I'm around people I know closely during the pandemic | Strongly disagree | Strongly agree |
| PbcCaOlder | Q11 | I believe I can keep at least 1 meter distance when communicating with older people during the pandemic | Strongly disagree | Strongly agree |
| PbcCaSign | Q12 | I believe I can keep at least 1 meter distance in crowded places even if there is no keep-a-distance marker | Strongly disagree | Strongly agree |
| PbcCaRoom | Q13 | I believe I can keep at least 1 meter distance in crowded places during the pandemic even if the places are tight/small | Strongly disagree | Strongly agree |
| PbcCaCrowd | Q14 | I believe I can keep at least 1 meter distance in crowded places during the pandemic even if there are too many people | Strongly disagree | Strongly agree |
| ***Perceived autonomy*** | | | | |
| PbcAuNeighbour | Q1 | How far you can control yourself to keep at least 1 meter distance when you are in your neighborhood during the pandemic | Very unlikely can control | Very likely can control |
| PbcAuShopping | Q2 | How far you can control yourself to keep at least 1 meter distance when shopping at the store/supermarket/market during the pandemic | Very unlikely can control | Very likely can control |
| PbcAuSport | Q3 | How far you can control yourself to keep at least 1 meter distance when exercising outside the house during the pandemic | Very unlikely can control | Very likely can control |
| PbcAuDailyActive | Q4 | How far you can control yourself to keep at least 1 meter distance while doing daily activities outdoors (e.g. work, school) during the pandemic | Very unlikely can control | Very likely can control |
| PbcAuKnown | Q5 | How far you can control yourself to keep at least 1 meter distance when you go to a familiar environment during the pandemic | Very unlikely can control | Very likely can control |
| PbcAuFamily | Q6 | How far you can control yourself to keep at least 1 meter distance when you meet your family during the pandemic | Very unlikely can control | Very likely can control |
| PbcAuFamily1 | Q7 | How far you can control yourself to keep at least 1 meter distance when visiting your family during the pandemic | Very unlikely can control | Very likely can control |
| PbcAuFriend | Q8 | How far you can control yourself to keep at least 1 meter distance when you meet your friends during the pandemic | Very unlikely can control | Very likely can control |
| PbcAuFriend1 | Q9 | How far you can control yourself to keep at least 1 meter distance when visiting your friends during the pandemic | Very unlikely can control | Very likely can control |
| PbcAuClosePerson | Q10 | How far you can control yourself to keep at least 1 meter distance when you are around people you know closely during the pandemic | Very unlikely can control | Very likely can control |
| PbcAuOlder | Q11 | How far you can control yourself to keep at least 1 meter distance when you talk to older people during the pandemic | Very unlikely can control | Very likely can control |
| PbcAuAsked | Q12 | How far you can control yourself to keep at least 1 meter distance when other people ask you to disregard social distancing during the pandemic | Very unlikely can control | Very likely can control |
| PbcAuRoom | Q13 | How far you can control yourself to keep at least 1 meter distance when you are in small/tight places | Very unlikely can control | Very likely can control |
| PbcAuCrowd | Q14 | How far you can control yourself to keep at least 1 meter distance when there are too many people at one place | Very unlikely can control | Very likely can control |
| ***Intention*** | | | | |
| IntProximity | Q1 | I intend to keep a distance 1 meter in crowded places during the pandemic | Strongly disagree | Strongly agree |
| IntInRush | Q2 | I intend to keep at least 1 meter distance in crowded places during the pandemic even if the places are small/tight | Strongly disagree | Strongly agree |
| IntTrouble | Q3 | I intend to keep at least 1 meter distance in crowded places during the pandemic even if it’s troublesome | Strongly disagree | Strongly agree |
| IntCost | Q4 | I intend to to keep at least 1 meter distance in crowded places during the pandemic even if I am in busy places | Strongly disagree | Strongly agree |
| ***General beliefs*** | | | | |
| GenBelIndoor | Q1 | Getting infected with corona virus by other people | Can only occur in a closed room (indoor) | Can occur in an open space (outdoor) or a closed room (indoor) |
| GenBelSymptomatic | Q2 | Getting infected with corona virus by other people | Can only occur when the person has symptoms | Can occur even though the person is asymptomatic |

Note: * unfavorable item: needs to be recoded
